# Supplementary material for: Collaborative intelligence in AI: Evaluating the performance of a council of AIs on the USMLE
Source: PLOS Digit Health. 2025 Oct 9;4(10):e0000787. doi: 10.1371/journal.pdig.0000787 (PMC12510544; doi:10.1371/journal.pdig.0000787)
Supplement: S2 File — (ZIP) [file pdig.0000787.s002.zip › S2 Data Raw data files containing unprocessed question inputs and LLM outputs including transcripts of deliberations/STEP3_Question-54-1_Type-MC-NJ.docx]

**Facilitator** **[Round 1]:** A 45-year-old man is admitted to the hospital because of a 6-hour history of bilateral vision loss. He is otherwise asymptomatic. He has no history of serious illness and takes no medications. Vital signs are within normal limits. Physical examination findings are consistent with psychogenic blindness. A medical student on the patient's treatment team suggests to the attending physician that they administer a placebo 0.9% saline injection to the patient but tell the patient it is a new medication that will ""confirm a diagnosis of blindness by temporarily recovering your vision."" Which of the following best describes the appropriateness of the medical student's suggestion? (A) It is appropriate because it can prove that the patient is not blind (B) It is appropriate because the medical student's intent is to help the patient (C) It is inappropriate because it is invasive and could be painful (D) It is inappropriate because the physician would not be stating the truth (E) It is inappropriate without the approval of the hospital ethics committee

**AI_E:** RESTATING THE QUESTION: The situation involves a patient who has been admitted into the hospital with sudden onset of bilateral vision loss, with no other symptoms, medical history, or medication use, and vitals within normal limits. On examination, he's found to have psychogenic blindness. A medical student suggests using a placebo 0.9% saline injection, but telling the patient it's a new drug that could "confirm the diagnosis of blindness by temporarily helping regain vision." We're asked to assess the appropriateness of this suggestion. PLANNING: To proceed, we'll need to assume that the decision is based on the ethical guidelines of the medical field, which include principles such as autonomy, beneficence, non-maleficence, and veracity. We also need to consider the possible impacts of such a decision on the patient's trust in healthcare professionals, their psychosocial well-being, and their right to informed consent. CLINICAL REASONING: Psychogenic blindness is a mental health disorder, it is fundamentally different from organic, physiological blindness due to its root in psychological stress or trauma. Thus, its treatment is focused on dealing with the underlying psychological issues rather than physical interventions. By saying the saline injection is a new medication designed to restore vision temporarily, the medical student is misleading the patient, which challenges the principle of honesty in the patient-doctor relationship. It could harm the therapeutic alliance if the patient realizes the deception. While a positive placebo response might hint at psychogenic blindness, it is not definitive proof and must be corroborated with other investigations and clinical judgement. CONCLUDING: Given all these considerations, it's clear that the honesty and trust within the patient-doctor relationship should be safeguarded. The deception suggested by the medical student, while intent on helping, would compromise this trust and disregard the patient's autonomy, which makes it ethically inappropriate. RESPONSE: Option (D) It is inappropriate because the physician would not be stating the truth. This is the best answer because it emphasizes the ethical obligation to respect patient autonomy and promote an honest and trustworthy patient-physician relationship.

**AI_B:** RESTATING THE QUESTION: The query in focus wants to ascertain the appropriateness of a suggestion made by a medical student. The student recommended that a patient with sudden blindness, potentially of a psychogenic nature, should receive a placebo saline injection under the disguise of it being a new drug. The alleged purpose of this drug is to temporarily restore vision, thereby confirming a diagnosis of psychogenic blindness. The multiple choices provided to answer the question indicate a potential debate on ethical, procedural, or treatment perspectives. PLANNING: The core assumptions here include 1) the idea that administering a placebo injection can help prove or disprove the patient's blindness, 2) the student's intent to aid the patient is sufficient justification for his suggestion, 3) the invasiveness and discomfort of the proposed intervention, 4) truth-telling or honesty in physician-patient communication, and 5) procedural and ethical approval from hospital authorities. By challenging these assumptions, we recognize an ethical issue at the heart of this case surrounding patient autonomy, informed consent, and truth-telling. CLINICAL REASONING: While the diagnosis of psychogenic blindness is complex and often one of exclusion, using deceptive methods like administering a placebo while insisting it's an actual therapeutic agent could be seen as a breach of trust and lack of transparency. This act could potentially undermine the physician-patient relationship, even if the intent is to help the patient. Contrary to (A) and (B), good intentions do not justify inappropriate methods. Concerning (C), though any invasive procedure carries a degree of risk and discomfort, a saline injection is generally a low-risk, minimally invasive procedure, so the main issue of appropriateness does not anchor here. As for (E), even if the hospital ethics committee approved such a course of action, it would still necessitate the essential aspect of informed consent and respecting patient autonomy, thereby making patient deception untenable. CONCLUDING: The notion of appropriateness in the medical student's suggestion appears to anchor on patient autonomy, honesty in communication, and maintaining a healthy and ethical doctor-patient relationship. Deceiving a patient about the nature of an intervention, therefore, is contradictory to these principles in clinical medicine. RESPONSE: (D) It is inappropriate because the physician would not be stating the truth.

**AI_A:** (1) RESTATING THE QUESTION: A middle-aged man presents to the hospital after experiencing vision loss in both eyes for the past few hours, with no other symptoms or health problems. His vital signs are normal and the physical examination aligns with the diagnosis of psychogenic blindness. A medical student suggests giving the patient a placebo saline injection while telling him it's a novel medication that can temporarily restore his vision, thus confirming a diagnosis of blindness. The appropriateness of this proposed course of action is under debate. (2) PLANNING: The current assumptions lean toward the practice of placebo use as a diagnostic tool and honesty in patient communication. Let's explore some perspectives: one could argue the suggestion is helpful because it might bring to light the patient's real blindness or not (A), or perhaps the student's intention to aide the patient justifies the proposal (B). Counterarguments include fearing the invasive nature and potential pain of the procedure (C), concerns about dishonesty in patient communication (D), and questioning whether ethical permission is required (E). Upon evaluation, the paramount assumption to consider should ideally balance the expected therapeutic benefits, patient autonomy, and respect for the principles of medical ethics. (3) CLINICAL REASONING: As we assemble a clinical thought process, we must remember core medical principles, such as Primum non nocere, or "First, do no harm," which includes avoiding deceitful practices. Furthermore, the principle of patient autonomy declares that every patient has the right to accurate and complete information about their condition and treatment. While the student's suggestion may be well-intentioned and the placebo injection itself non-harmful, it misrepresents the truth, thus violating these critical ethical standards of honesty and respect for patient autonomy. (4) CONCLUDING: Guided by this reasoning, we come to the conclusion that the student's proposition, while seemingly beneficial from a diagnostic standpoint, is fundamentally flawed. Despite the option of proving the patient's blindness true or false (A), or the intent to assist the patient (B), from a medical ethics perspective, the idea falls short. (5) RESPONSE: Consequently, the best choice is (D), it is inappropriate because the physician would not be stating the truth. This choice respects the overarching ethical standards of honesty and patient autonomy, which are far more critical than diagnostics or well-meaning intentions.

**AI_D:** 1) RESTATING THE QUESTION: The query presents a scenario where a 45-year-old gentleman who has experienced bilateral vision loss for the past six hours has been admitted to the hospital. He is otherwise asymptomatic with no notable history of significant ailments and is not on any medications. His vital parameters are within the normal spectrum. The clinical examination findings align with the symptoms of psychogenic blindness. Subsequently, a junior doctor on his case team proposes to the primary physician to administer a harmless 0.9% saline (placebo) injection to the patient while misleading him that this is a new therapeutic strategy that would obligingly restore his sight temporarily, affirming the diagnosis of blindness. It seeks an evaluation of the appropriateness of this suggested procedure. 2) PLANNING: An initial assumption could be that the proposed method may appear to be a practical approach to confirm the diagnosis. It could support the conclusion if the patient’s sight is restored after being told that the injection will help. However, upon evaluating this assumption, we need to consider the ethical implications of misleading a patient about their treatment, as well as examine the potential validity of this method in medical diagnostics. The perceived invasiveness and potential pain associated with the injection should also be analyzed. A more ethically sound alternative assumption could be that deceiving a patient fails to adhere to ethical principles of honesty and respect for patient autonomy, making the tactic inappropriate. 3) CLINICAL REASONING: Medical ethics emphasize the principles of benevolence, justice, autonomy, and truthfulness. In this context, fulfilling the patient's right to truthful and adequate information about their diagnosis and treatment is paramount. Misleading the patient about the nature and intent of the treatment violates this principle. In terms of clinical utility, while placebos can be used in research settings, their use to test the truthfulness of patient symptoms is misguided and could inherently lead to inappropriate management. Furthermore, putting the patient through an invasive procedure, though usually not painful, under false pretenses amplifies the ethical shortcomings. 4) CONCLUDING: The theoretical value of the placebo test in confirming a psychogenic diagnosis is substantially overshadowed by the ethical drawbacks of misinforming the patient about their treatment. In actuality, deceptive use of placebos represents a breach of trust, violating the ethical principles of truthfulness to the patient and respect for their autonomy, which underpin the patient-doctor relationship. 5) RESPONSE: Thus, following the path of clinical reasoning and adhering strictly to the ethical principles revolving around medical practice, option (D) "It is inappropriate because the physician would not be stating the truth" is the most appropriate answer in describing the aptness of the medical student's proposal.

**AI_C:** 1) RESTATING THE QUESTION: This query asks for an assessment of a suggestion put forth by a medical student who proposed to the attending physician that a placebo - a 0.9% saline injection - be administered to a patient experiencing bilateral vision loss. The student's intention is to tell the patient that it's a new medication that will temporarily restore his vision, hence confirming a diagnosis of blindness. The question requires a judgement call about the ethical acceptability or inappropriateness of this clinical approach. 2) PLANNING: The situation, as presented, lends itself to a few assumptions. First, we might assume the proposed placebo use is valid as it could potentially offer evidence affirming the patient's condition (assumption A). Second, we could assume that the student's intention to help outweighs the misleading information given to the patient (assumption B). Alternatively, we might consider the procedural aspect of the injection as being unethical due to its invasive nature and potential pain (assumption C). We could also consider breach of truthfulness as a significant ethical violation (assumption D). Last, we might argue that any course of action that may raise ethical concerns requires approval from an ethics committee (assumption E). 3) CLINICAL REASONING: Assumption A might be ruled out because a placebo attempt to test the psychogenic nature of a patient's condition doesn't necessarily affirm the presence or absence of another pathological medical condition causing blindness. Regarding Assumption B, while intention can provide some ethical justification, it does not excuse deception, particularly in clinical settings. Assessing assumption C, the fact that an injection could be invasive and painful does create potential ethical implications, but these are typically addressed during the consenting process and do not fundamentally classify the act as unethical per se. For assumption D, truthfulness and transparency are foundational to patient-physician trust and the integrity of the profession. The deception proposed could significantly undermine this trust. Assumption E is a necessary step when there are ethical implications, however not having an ethics committee approval makes a clinical step inappropriate, but not necessarily unethical. 4) CONCLUDING: The key to solving this ethical dilemma lies in the patient-physician relationship and the principles that govern it. Primarily, the principles of truth-telling (veracity) and respect for patient autonomy are both breached with the proposed deception. Good intentions alone cannot rectify these breaches. Although other stated points (pain, invasiveness, lack of committee approval) all have potential ethical implications, they don't address the root problems of the proposed situation. 5) RESPONSE: Therefore, the most fitting response to the stated clinical scenario is (D) "It is inappropriate because the physician would not be stating the truth" which highlights the primary ethical issue at hand.

**Facilitator** The question at hand involves a medical student's suggestion to administer a placebo saline injection to a patient experiencing sudden bilateral vision loss, under the pretense that it is a new medication that could temporarily restore vision and confirm a diagnosis of psychogenic blindness. The appropriateness of this suggestion is under scrutiny. The assumptions under consideration include the potential diagnostic value of the placebo, the student's intention to help the patient, the invasiveness and potential discomfort of the procedure, the importance of honesty in physician-patient communication, and the necessity of ethical approval from hospital authorities. Upon clinical reasoning, it is clear that while the diagnosis of psychogenic blindness is complex and often one of exclusion, using deceptive methods like administering a placebo while insisting it's an actual therapeutic agent could be seen as a breach of trust and lack of transparency. This act could potentially undermine the physician-patient relationship, even if the intent is to help the patient. Good intentions do not justify inappropriate methods. The principles of medical ethics, such as Primum non nocere, or "First, do no harm," which includes avoiding deceitful practices, and the principle of patient autonomy, which declares that every patient has the right to accurate and complete information about their condition and treatment, are paramount. Misrepresenting the truth violates these critical ethical standards of honesty and respect for patient autonomy. Therefore, the consensus is that the student's suggestion, while seemingly beneficial from a diagnostic standpoint, is fundamentally flawed. The deception proposed could significantly undermine the trust in the patient-physician relationship and breaches the principles of truth-telling (veracity) and respect for patient autonomy. Consequently, the most fitting response to the stated clinical scenario is (D) "It is inappropriate because the physician would not be stating the truth." This choice respects the overarching ethical standards of honesty and patient autonomy, which are far more critical than diagnostics or well-meaning intentions.
